# Beyond polarization: macrophage senescence in immunoregulation and cancer therapy

**DOI:** 10.7150/ijbs.115921

**Published:** 2025-06-23

**Authors:** Mei Song, Lifen Zhang, Xiaofeng Dai, Shiliang Ji, Jiayin Shen, Weiling He

**Affiliations:** 1 Department of Gastrointestinal Surgery, The First Affiliated Hospital of Sun Yat-sen University, Sun Yat-sen University, Guangzhou, Guangdong 510080, China.; 2 Department of Gastrointestinal Surgery, Xiang'an Hospital of Xiamen University, School of Medicine, Xiamen University, Xiamen, Fujian 361000, China.; 3 National Local Joint Engineering Research Center for Precision Surgery & Regenerative Medicine, Shaanxi Provincial Center for Regenerative Medicine and Surgical Engineering, First Affiliated Hospital of Xi'an Jiaotong University, Xi'an, Shaanxi 710061, China.; 4 Suzhou Research Center of Medical School, Suzhou Hospital, Affiliated Hospital of Medical School, Nanjing University, Suzhou 215000, China; 5 National Clinical Research Center, Shenzhen Third People's Hospital, Shenzhen, Guangdong 518112, China.

**Keywords:** age-related diseases, cancer microenvironment, macrophage senescence, senescence-associated secretory phenotype (SASP), therapeutic targeting

## Abstract

Cancer incidence is increasing globally, presenting significant health challenges due to its severe impact on morbidity and mortality. As a disease closely linked to aging, the prevalence of cancer is expected to increase with increasing age, underscoring the need for comprehensive research into its mechanisms and treatments. Macrophages, which are central to the immune system, play a paradoxical dual role in cancer progression. While they can suppress tumor growth, tumor-associated macrophages (TAMs) frequently facilitate tumor development and metastasis, a complexity that is further intricate by the aging process. As macrophages transition into senescent cells, they undergo changes, including shifts in cytokine profiles, reduced phagocytic activity, and altered metabolism. These senescent macrophages contribute to cancer progression by creating an immunosuppressive environment, promoting angiogenesis, and supporting tumor invasion. This review explores the intricate functions of senescent macrophages in cancer, highlighting their implications for tumor biology and their potential as therapeutic targets. We discuss strategies to manipulate senescent macrophages to enhance current cancer therapies, emphasizing the importance of understanding their mechanisms to advance cancer treatment.

## 1. Introduction

Globally, cancer ranks as a primary contributor to death with its occurrence increasing as a result of elements such as older demographic and personal lifestyle decisions 1-5. The impact on health is profound, with patients frequently enduring a spectrum of side effects from both the malignancy and its treatments, such as pain, exhaustion, and psychological turmoil 6. The World Health Organization predicts a significant increase in cancer cases, from approximately 12.7 million in 2008 to 35 million by 2050, with a corresponding doubling of cancer-related deaths 7. In China, the elderly population is disproportionately impacted, with a substantial proportion of cancer cases and deaths occurring in those over 80 years of age 8. These alarming projections and the growing burden of cancer highlight the urgent need for advancements in cancer therapy. Current treatments, however, face significant challenges, including drug resistance, immune evasion, and treatment recurrence. Addressing these gaps is crucial for improving the therapeutic outcomes and quality of life of cancer patients.

As individuals age, the frequency and death rates of different tumors increase, categorizing malignant tumors as age-related degenerative diseases 9. Senescence plays multifaceted and often paradoxical roles in cancer progression. Cellular senescence, characterized by a permanent halt in the cell cycle, acts as a mechanism that suppresses tumors by inhibiting the growth of cells that are either damaged or activated by oncogenes 10, 11. Nonetheless, these senescent cells can also promote tumor development through multiple pathways, particularly via the senescence-associated secretory phenotype (SASP), which modifies the tumor microenvironment (TME) and aids in evading immune detection 12-16. Immunosenescence refers to the decline in immune function associated with aging, and this process is further complicated by age-related alterations in the immune system 17-19. In older patients, senescent stromal cells may fill the TME, promoting an immunosuppressive setting that allows tumors to escape immune surveillance and diminishes the effectiveness of cancer treatments, such as immunotherapy 20. Macrophage play crucial roles within the immune system and have dual functions in cancer 21-25. On the one hand, these cells can promote antitumor responses by identifying and destroying cancerous cells via phagocytosis and the release of proinflammatory cytokines. Conversely, tumor-associated macrophages (TAMs), which typically exhibit an M2-like phenotype, may increase tumor growth and metastasis through promoting angiogenesis, suppressing antitumor immune responses, and enhancing tumor cell invasion and migration. Macrophage senescence, characterized by stable cell cycle arrest, altered cytokine production, and metabolic changes, significantly impacts the interplay with tumor cells, contributing to an immunosuppressive TME that promotes cancer growth and immune evasion 26, 27.

Macrophage senescence plays a pivotal role in cancer biology, making it imperative to explore the underlying mechanisms that drive this process. A deeper understanding of these mechanisms could lead to the development of novel therapeutic strategies aimed specifically at senescent macrophages. By targeting these cells, it may be possible to increase the effectiveness of existing cancer treatments, potentially improving patient outcomes and paving the way for more effective treatment options in oncology. This review provides an in-depth examination of the function of senescent macrophages within the TME, explores their implications for cancer biology, and discusses the potential for targeting macrophage senescence in therapeutic strategies.

## 2. Characteristics of Macrophage Senescence

### 2.1 Definition and biomarkers of macrophage senescence

**Figure 1:**
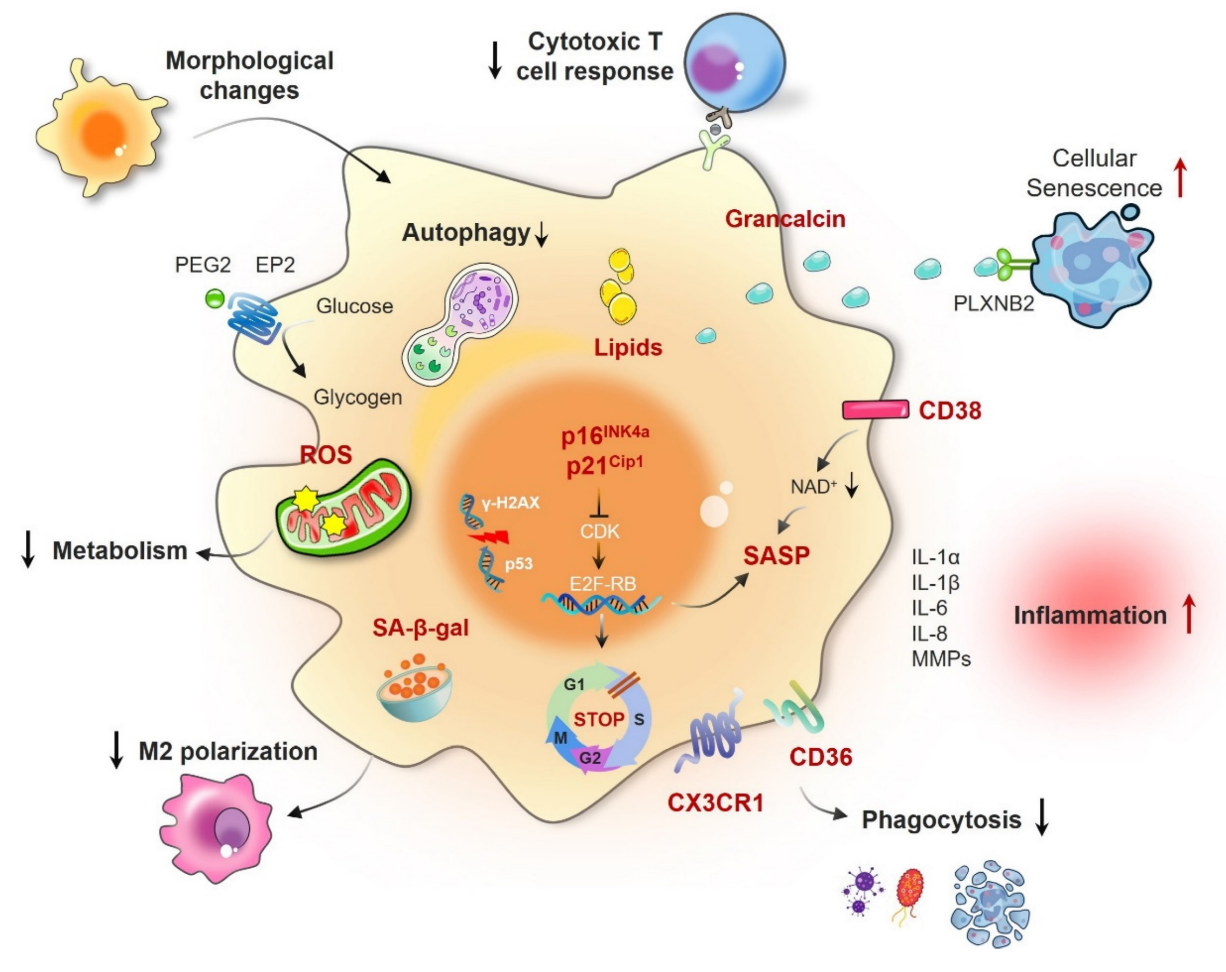
** Functional and cellular changes in senescent macrophages.** Macrophage senescence is a state of permanent cell cycle arrest characterized by increased activity of senescence-associated beta-galactosidase (SA-β-gal); the overexpression of senescence biomarkers such as p16INK4a, p21CIP1, p53, CD38, and CX3CR1; and the secretion of senescence-associated secretory phenotype (SASP) factors, including IL-1, IL-6, IL-8, and matrix metalloproteinases (MMPs). These changes are associated with morphological alterations such as increased cell size and nuclear deformities, metabolic reprogramming leading to altered energy production, compromised polarization capacity resulting in an inability to effectively switch between the M1 and M2 phenotypes, and impaired phagocytic function with a reduced capacity to engulf pathogens and cellular debris.

As essential elements of the immune system, macrophages are tasked with eliminating pathogens and cellular debris, and they also serve critical functions in inflammation and tissue repair. However, under conditions of aging or certain pathologies, these cells may undergo senescence, a state defined by irreversible growth arrest 28. Senescent macrophages display functional and phenotypic alterations that compromise their immune surveillance and clearance capabilities. The identification of key biomarkers is essential for uncovering the characteristics and mechanisms underlying macrophage senescence, thereby enhancing our understanding of its implications in disease progression (Fig. 1).

#### 2.1.1 Senescence-associated beta-galactosidase (SA-β-Gal)

SA-β-Gal is a well-established biomarker for cellular senescence, distinguished by its heightened enzymatic activity at pH 6. This characteristic enables histochemical detection of senescent cells both *in vitro* and *in vivo* 29. Research has indicated that in older mice, the buildup of macrophages that express SA-β-Gal is linked to chronic systemic inflammation related to aging 30. Subsequent investigations into the expression of SA-β-Gal in macrophages revealed that this marker is subject to reversible modulation by physiological stimuli known to drive macrophage polarization 31. Yang et al*.* investigated the senescence of macrophages within gastric cancer organoids induced by Aurora kinase inhibitors and reported that these macrophages exhibited senescent characteristics, including increased SA-β-Gal expression 32. Moreover, senescent cancer cells are capable of recruiting and inducing M2 polarization by secreting monocyte chemoattractant protein-1 (MCP-1/CCL2), thereby suppressing their innate immune response to cancer cells. In summary, SA-β-Gal serves not only as a marker of senescent cells but also as a reversible marker of macrophage polarization.

#### 2.1.2 p16INK4a and p21Cip1

The cell cycle is regulated primarily by cyclin-dependent kinase inhibitors (CDKIs), such as p16INK4a and p21Cip1, which are highly expressed in senescent macrophages and serve as indicators of cellular aging. p16INK4a interacts with CDK4 or CDK6 to inhibit the formation and activity of cyclin D-CDK complexes, while p21Cip1 binds to cyclin E-CDK2 complexes, directly suppressing their activity. This inhibition prevents the phosphorylation of the retinoblastoma protein (Rb), maintaining the hypophosphorylation of Rb in cells in the G1 phase, thus halting their transition into the S phase and suppressing cell proliferation 33. The increase in these CDK inhibitors within macrophages not only indicates a reaction to cellular stress but also reflects the cell's choice to enter a state of senescence. According to Smith and colleagues, macrophages that are cultured for a duration of 7--14 days display characteristics similar to senescence, characterized by diminished proliferation and increased concentrations of p16INK4a and p21Cip, both of which play a role in chronic inflammation and age-associated diseases 34. Other studies also corroborated the roles of p16INK4a and p21Cip1 in the aging of RAW264.7 macrophages 31, 35, 36.

#### 2.1.3 Senescence-associated secretory phenotype (SASP)

Cellular senescence is defined by the SASP, which encompasses diverse secretory profiles containing proinflammatory cytokines, chemokines, growth factors, and matrix metalloproteinases (MMPs). This specific phenotype plays an essential role in the interaction between senescent cells and their surrounding environment 37-39. Proinflammatory cytokines, including interleukin (IL)-6, which are components of the SASP produced by aging macrophages, significantly contribute to the chronic inflammation observed in older tissues and several diseases linked to aging 40. Recently, Prieto et al*.* reported that senescent alveolar macrophages accumulate early in the development of KRAS-driven lung cancer in a murine model and play a role in the process of tumor formation. These macrophages exhibit increased levels of p16INK4a and CXCR1, which can inhibit the responses of cytotoxic T cells. Removing these senescent macrophages in mice reduces adenoma development and progression, highlighting their role in promoting tumorigenesis 14. In another study focusing on lumbar spine instability and aging in male mice, IL-10 was identified as the primary elevated cytokine in senescent-like macrophages. The removal of the cdkn2a (p16) gene in macrophages was shown to improve lumbar spine instability and mitigate angiogenesis and sclerosis in the endplates associated with aging 41. Together, the complexity of the SASP in senescent macrophages underscores their multifunctional nature in disease pathogenesis 42.

#### 2.1.4 Reactive oxygen species (ROS)

The theory of aging related to free radicals, first introduced by Denham Harman in 1956, suggests that the unavoidable generation of ROS during metabolic processes leads to oxidative harm to DNA, proteins, and lipids. This accumulation of damage is considered one of the main factors contributing to aging 43. Nonetheless, subsequent experimental and clinical research has questioned this theory, as high levels of antioxidants have not been shown to have any effect on aging or age-related diseases. Mice lacking antioxidant enzymes such as manganese superoxide dismutase (MnSOD) and glutathione peroxidase-1 (Gpx-1) do not have a shorter lifespan 44. Even with these difficulties faced by the free radical theory of aging, oxidative stress continues to play a significant role in the aging process and in numerous age-related diseases. Recent studies have suggested that ROS not only serve as biomarkers of cellular aging but also critically regulate cellular senescence. Studies on squamous cell carcinoma of the head and neck (HNSCC) have demonstrated that persistent exposure to low concentrations of ROS results in prolonged expression of p21 in HNSCC cells exhibiting partially functional TP53, ultimately causing cellular senescence 45. Additionally, a different study demonstrated that UP446, which is a naturally occurring flavonoid mixture, displays notable antioxidant properties and immunomodulatory effects in mice subjected to accelerated aging and chemically induced immune suppression. UP446 enhances the immune response by activating both innate and adaptive components, increasing antioxidant levels, maintaining essential immune organs such as the thymus, and decreasing NF-κB activity 46. Together, these findings clarify the significant function of ROS in the aging of the immune system and suggest possible therapeutic approaches for addressing age-related changes by modulating ROS concentrations.

#### 2.1.5 Specific markers

The senescence of macrophages is characterized by the expression of particular proteins, such as grancalcin (GCA), C-X3-C motif chemokine receptor 1 (CX3CR1), and cluster of differentiation 38 (CD38), which play vital roles in understanding how macrophages contribute to the aging process. Research by Zou et al*.* revealed that GCA, which is secreted by aging macrophages, triggers senescence in skeletal stem/progenitor cells and hinders the healing of fractures. Specifically, GCA interacts with the plexin-B2 receptor, resulting in the activation of Arg2, which causes mitochondrial dysfunction and ultimately leads to cellular senescence 47. During the aging process, there is an increase in the secretion of GCA, which is associated with the accumulation of proinflammatory and senescent immune cells, including macrophages and neutrophils, in the bone marrow. Genetic deletion of the GCA gene in these cells has been shown to delay skeletal aging in mice 48.

CD38 is a transmembrane protein that catalyzes the conversion of nicotinamide adenine dinucleotide (NAD^+^) to cyclic ADP-ribose (cADPR), ADP-ribose (ADPR), and nicotinamide (NAM), affecting vital cellular signaling pathways 49. Covarrubias et al*.* noted the buildup of proinflammatory M1-like macrophages within metabolic tissues, including visceral white adipose tissue and the liver, in the context of aging and acute inflammation. These M1-like macrophages are distinguished by increased CD38 expression and increased NADase activity, which play a role in lowering tissue NAD levels. In addition, senescent cells that progressively accumulate with age secrete SASP factors to facilitate macrophage proliferation and CD38 expression 50. Consequently, the activation of CD38 is correlated with increased NADase activity in macrophages, as well as the buildup of senescent cells and factors related to SASP factors.

The chemokine receptor CX3CR1, which is a key marker for tissue-resident macrophages, is vital for modulating their recruitment, activation, and functional capabilities. In the context of obesity and metabolic disorders, CX3CR1-positive macrophages interact with adipose-derived stem cells to maintain metabolic health. In particular, in visceral adipose tissue, CX3CR1*^hi^* macrophages support healthy tissue expansion by inhibiting adipose-derived stem cell senescence, promoting metabolic adaptation. However, this functionality is compromised in aged mice, leading to adipose stem cell aging and a decline in metabolic fitness 51. The importance of CX3CR1 in the aging of macrophages is additionally highlighted by research investigating muscle recovery after pneumonia caused by the influenza A virus. In older mice, the recovery of skeletal muscle is impaired due to inadequate macrophage expansion, diminished expression of MHC II, and a reduction in the proliferation of muscle satellite cells 52.

### 2.2 Physiological and pathological characteristics of macrophage senescence

As crucial elements of the immune system, macrophages play a key role in removing pathogens, deceased cells, and cellular waste. However, with age or under certain pathological conditions, macrophages may undergo senescence, resulting in unique physiological and pathological characteristics (Fig. 1 and Table 1).

#### 2.2.1 Changes in cellular function

Macrophages are essential for immune defense, pathogen clearance, and tissue repair, with phagocytosis being a key mechanism for these functions. Linehan and colleagues reported a notable decrease in the phagocytic ability of peritoneal macrophages with age, which was observed both *in vitro* and *in vivo* 53. This study revealed that even when young macrophages are introduced into the peritoneum of aged mice, their phagocytic function is compromised, suggesting that potential age-related extrinsic factors within the aged peritoneal microenvironment may contribute to this decline. The study also revealed an increase in T, B1 and B2 cells and in IL-10 production by B cells in aged mice, which may be correlated with the compromised phagocytic function of macrophages. Within the scope of *Streptococcus pneumoniae* infection, bone marrow-derived macrophages (BMDMs) from young mice efficiently employ LC3-associated phagocytosis to target bacterial vesicles. In contrast, BMDMs isolated from older mice exhibit shortcomings in this mechanism and in their ability to kill bacteria, along with generating elevated levels of proinflammatory cytokines 54. Conversely, Rymut et al*.* focused on the decrease in the capacity of macrophages to clear apoptotic cells related to aging. They reported that aging exacerbated pulmonary inflammation after remote limb ischemia‒reperfusion (I/R) injury, which was correlated with reduced MerTK levels on macrophages 55. These results indicate that with the aging of macrophages, their capacity to phagocytose pathogens and eliminate apoptotic cells notably deteriorates. In older animals, increased synthesis of proinflammatory cytokines, including tumor necrosis factor-α (TNF-α), IL-1α, and IL-6, was observed in macrophages, which could lead to an imbalance in inflammatory responses 29, 56. Such changes in macrophage functionality might significantly affect the vulnerability of elderly individuals and their reactions to infections.

#### 2.2.2 Metabolic features

Senescent macrophages undergo substantial changes in their metabolic profiles, typically characterized by a reduction in overall metabolic activity that could greatly impact their viability and functionality 29. During the transition to senescence, these macrophages experience a decrease in their basal metabolic rate and a decrease in the efficiency of energy-producing pathways, including glycolysis and oxidative phosphorylation. This metabolic shift can impair the essential functions of macrophages in immune surveillance, pathogen clearance, and tissue repair, potentially leading to a compromised immune response in aged individuals.

Research has indicated that the levels of nicotinamide adenine dinucleotide phosphate (NADPH) oxidase 4 (NOX4), an enzyme-linked to cellular stress, increase in aging macrophages, which aligns with mitochondrial dysfunction, inflammation, and the development of atherosclerosis 57. The signaling pathway of prostaglandin E2 and epidermal growth factor receptor 2 (PGE2-EP2) is crucial for modulating metabolism and inflammatory reactions in myeloid cells. In aging macrophages, the action of PGE2 via the EP2 receptor encourages the storage of glucose as glycogen, which decreases glucose flow and mitochondrial respiration. This condition results in a shortage of energy, contributing to maladaptive inflammation and a decline in cognitive function 58. Furthermore, metabolic alterations in senescent macrophages can result in the accumulation of ROS within the cells. ROS are byproducts of normal metabolic processes and can become detrimental when present in excessive amounts, causing oxidative stress and damaging cellular components such as proteins and DNA 59. In diabetes, persistent hyperglycemia and oxidative stress conditions increase ROS levels in macrophages, which not only amplify inflammatory responses but also accelerate macrophage aging. Senescent macrophages exhibit impaired immune functions, releasing proinflammatory cytokines and chemokines that further exacerbate oxidative stress and inflammation, creating a vicious cycle of oxidation‒inflammation‒aging 57, 60.

#### 2.2.3 Morphological and phenotypic features

Aging causes substantial morphological and phenotypic changes in macrophages, contributing to their functional decline. Morphologically, senescent macrophages display enlarged cell sizes and nuclear deformities, along with alterations in cellular organelles, especially concerning the functionality and structure of the endoplasmic reticulum and mitochondria. Moreover, these senescent macrophages exhibit significant differences in their biochemical phenotypes, which are linked to various biological functions 61. By employing synchrotron radiation Fourier transform infrared microspectroscopy, scientists have identified biochemical phenotypes as well as differences in phenotypes at the level of individual macrophages, revealing dynamic changes in lipids and proteins, particularly cholesterol esters, as a result of macrophage senescence. Additionally, the morphological alterations, such as enlarged cell size and reduced number of pseudopodia, observed in senescent macrophages are linked to the activation of the SASP 62. Furthermore, senescent macrophages upregulate proteins such as p53, p21, and γ-H2A. X, which are indicative of DNA damage and repair mechanisms. RNA sequencing and metabolomic analysis have revealed significant alterations in pathways associated with inflammation, lipid metabolism, and insulin signaling in senescent macrophages, with increased lipid accumulation and insulin resistance 63. These modifications not only impact aging macrophages directly but also induce systemic changes by influencing the microenvironment via the secretion of soluble factors and extracellular vesicles, which in turn accelerates the progression of aging and associated diseases.

#### 2.2.4 Role in chronic diseases

Obesity and the aging process are significant factors in metabolic syndrome, as they influence the body's inflammatory state and immune capabilities, thereby facilitating the onset and progression of diseases. Lumeng et al*.* conducted a thorough investigation into the different subtypes of adipose tissue macrophages (ATMs) in both young and aged mice and revealed that aging is associated with qualitative changes in ATMs, leading to a proinflammatory environment, with reduced resident type 2 (M2) ATMs and increased CD206^-^CD11c^-^ (double negative) ATMs 64. Rabhi et al*.* reported that early adipocyte progenitor cells expressing both platelet-derived growth factor receptor (PDGFR) α and β are the major contributors to extracellular matrix deposition. The fibrotic process is facilitated by macrophages that have entered a senescent state. These macrophages develop SASP and gradually lose their ability to perform phagocytosis effectively. In the fibrotic environment, osteopontin produced by senescent macrophages promotes the proliferation of progenitor cells and fibrotic gene expression and inhibits adipogenesis 65. Nagai et al*.* established a direct connection between senescent macrophages and age-related macular degeneration (AMD). Research has revealed that mice fed a high-fat diet accumulate oxidized low-density lipoprotein (ox-LDL) within macrophages via the renin‒angiotensin system, which in turn facilitates the progression of AMD. The macrophages laden with ox-LDL expressed inflammatory cytokines and attacked the retinal pigment epithelium, leading to visual impairment. Furthermore, senescent macrophages not only increase inflammation and fibrosis in adipose tissue but also influence insulin sensitivity and metabolic health 66. The buildup and functional alterations of senescent macrophages could signify crucial links among obesity, inflammation, and metabolic disorders.

Moreover, senescent macrophages influence the TME by manipulating cancer cell behavior. A range of proinflammatory cytokines and growth factors are released by these cells, establishing a conducive environment that might promote the survival, proliferation, and metastasis of tumor cells. Studies have suggested that the infiltration of macrophages, such as those in breast, lung, liver, pancreatic, and colorectal cancer, into the TME increases with age, functioning in tumor progression and therapeutic response 14, 67-72. Elderly individuals presented a greater presence of CD68^+^/CD206^+^ cells within tumor tissues than younger individuals did. Furthermore, TAMs sourced from older mice were more effective at facilitating the proliferation of CT26 tumor cells, leading to the development of larger tumors in aged mice than in their younger counterparts. In addition, aged TAMs exhibit increased expression of genes associated with tumor promotion but decreased expression of genes associated with tumor suppression 73. The relationship between senescent macrophages and tumor cells can be especially harmful, potentially enhancing cancer aggressiveness and contributing to resistance to treatment options.

#### 2.2.5 Cell‒cell interactions

Aging macrophages show notable alterations in how they interact with various cell types, including T cells and stem cells, profoundly influencing their functionality and the overall tumor immune response. Studies have shown that with age, macrophages can become less proficient in stimulating antitumor T-cell responses. For example, in elderly mice, TAMs often predominate in the TME, fostering an immunosuppressive milieu that inhibits T-cell activity. This is particularly evident in studies demonstrating that an elevated presence of immunosuppressive, senescent macrophages in tumors can diminish the proliferation and activation of tumor-specific cytotoxic T lymphocytes (CTLs) 74, 75. Furthermore, older macrophages frequently show increased secretion of proinflammatory cytokines, including IL-6 and TNF-α, which play a role in a persistent inflammatory condition referred to as “inflammaging.” Such ongoing inflammation results in an imbalanced immune response, disrupting the equilibrium between proinflammatory and anti-inflammatory signals and ultimately influencing T-cell activity and the immune response to tumors 76. In addition to their interactions with T cells, senescent macrophages also engage with stem cells, particularly mesenchymal stem cells (MSCs). Aging impairs the number and function of MSCs, reducing their capacity to facilitate tissue repair. This impairment can result in delayed resolution of inflammation and a reduced capacity for tissue regeneration, which are pivotal for controlling tumor growth and responding to treatment 74, 77. The interaction between senescent macrophages and stem cells can also affect their polarization. For example, aged MSCs may fail to effectively induce the polarization of inflammatory macrophages (M1) toward an anti-inflammatory phenotype (M2), potentially worsening inflammation and supporting tumor progression 76, 78.

## 3. Mechanisms of Macrophage Senescence

**Figure 2:**
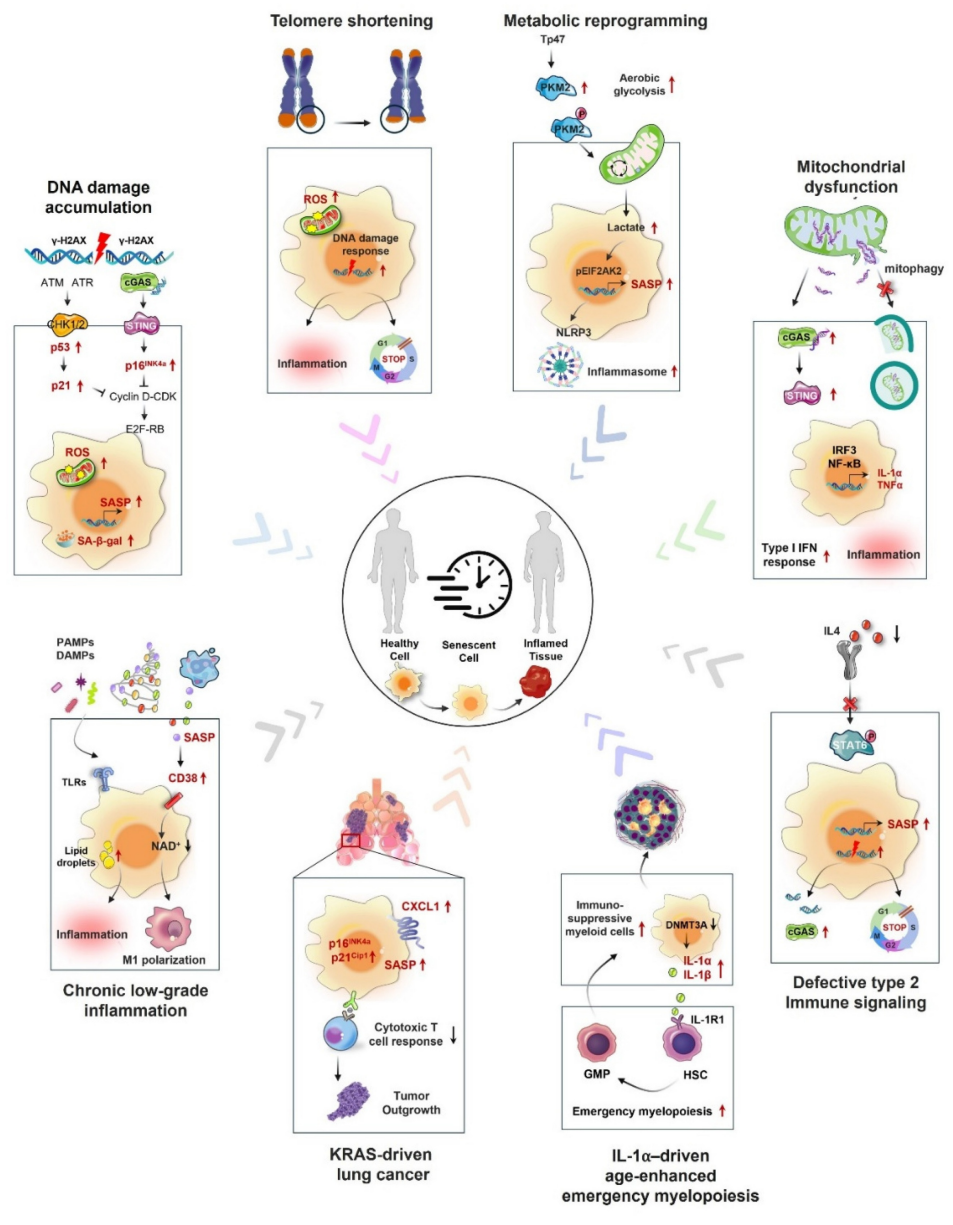
** Mechanisms of macrophage senescence in cancer.** Macrophage senescence involves a complex interplay of various mechanisms, including genomic instability leading to DNA damage response activation, oncogene mutations that trigger cell cycle arrest, telomere attrition causing replicative senescence, mitochondrial dysfunction resulting in increased reactive oxygen species (ROS) production, metabolic reprogramming shifting toward glycolysis, altered intracellular communication with disrupted signaling pathways, chronic inflammation induced by persistent SASP factor secretion, and defective type 2 immune signaling characterized by impaired anti-inflammatory responses. These mechanisms collectively contribute to the development and progression of cancer by creating an immunosuppressive TME and promoting tumor cell survival and proliferation.

The processes that contribute to macrophage aging involve a diverse range of cellular and molecular alterations, which can be affected by both internal and external factors (Fig. 2).

### 3.1 Precipitating factors of macrophage senescence

Macrophage senescence is a complicated process shaped by numerous triggering factors, such as genetic mutations, chemical exposures, inflammatory factors, cholesterol-mediated metabolic changes, SASP factors, and cell‒cell interactions. Mutations in genes such as KRAS play crucial roles in the aging of macrophages. These mutations disrupt intracellular signaling pathways, leading to functional changes and the onset of senescence 14, 68. Inflammatory factors, especially those from the IL-1 family, are significantly linked to cellular senescence. In aged mice, an increase in emergency myelopoiesis leads to the accumulation of myeloid progenitor-like cells in lung tumors. These cells, mainly macrophages, secrete IL-1α, which initiates a heightened myeloid response that promotes immunosuppression. Targeting this pathway with anti-IL-1α antibodies or the IL-1 receptor 1 (IL-1R1) blocker anakinra at the onset of tumor formation not only retards tumor progression but also restores myelopoiesis in older mice. Moreover, in human lung cancer lesions, an enrichment of IL-1α-expressing monocyte-derived macrophages is correlated with aging, poorer survival, and cancer recurrence 71. Cholesterol-mediated NAD^+^ depletion is another factor contributing to macrophage senescence. Cholesterol accumulation in macrophages promotes aging and is associated with key features of age-related macular degeneration, such as the accumulation of subretinal lipids. This metabolic shift may lead to a decline in cellular functions and the emergence of senescence markers 79. Age-related changes in collagen and matrix components prompt macrophage senescence. The accumulation of these components can impact macrophage functions, driving them into a senescent phase. These changes affect not only the metabolism and signal transduction of macrophages but also their responsiveness to external stimuli 80. Cell‒cell interactions, particularly during tissue damage or inflammation, can also promote macrophage senescence. For example, IL-6 derived from tumor cells can trigger senescence in macrophages, consequently influencing the TME 26.

### 3.2 Defective type 2 immune signaling pathway

Recent research has illuminated the importance of type 2 cytokine signaling in the modulation of both macrophage senescence and aging in organisms. Findings from these investigations revealed a reduction in the levels of essential elements of the IL-4 signaling cascade, including interleukin 4 receptor (IL4R), interleukin 13 receptor alpha 2 (IL13Rα1), and signal transducer and activator of transcription 6 (STAT6), as age increased in both mice and humans. This reduction was associated with increased levels of the DNA damage indicator p53. In the absence of STAT6, nuclear DNA leaks into the cytoplasm of macrophages, activating the cyclic GMP‒AMP synthase (cGAS)-stimulator of interferon genes (STING) pathway, which further promotes macrophage senescence and inflammation 35. Additionally, a different study revealed that the activation of the IL-4 signaling pathway diminished the levels of SASP factors and markers of cellular senescence 81. Overall, the type 2 cytokine signaling pathway, particularly the IL-4-STAT6 axis, is a critical regulator of the preservation of macrophage function, prevention of cellular senescence, and mitigation of aging-related processes.

### 3.3 Chronic low-grade inflammation (inflammaging)

Chronic low-level inflammation, often referred to as “inflammaging,” represents a key characteristic of the aging process that is linked to various diseases associated with aging. This phenomenon features a continual state of systemic inflammation, typified by increased concentrations of proinflammatory cytokines, including IL-6 and TNF-α, among older adults. These cytokines are essential for promoting the aging of immune cells, especially macrophages 78. As aging progresses, macrophages often exhibit increased expression of inflammatory cytokines and markers, including CD38. This polarization is influenced by SASP factors, which can disrupt normal cellular functions and promote further inflammation, creating a feedback loop that exacerbates tissue damage and dysfunction 50, 82. The processes that govern macrophage aging in the setting of persistent inflammation involve various pathways. A significant element is the activation of pattern recognition receptors (PRRs), including Toll-like receptors (TLRs), which identify pathogen-associated molecular patterns (PAMPs) and internal signals from injured tissues. The activation of TLRs triggers the release of proinflammatory cytokines, potentially amplifying macrophage activation and fostering chronic inflammation 78, 83. Moreover, during inflammation, the metabolic reprogramming of macrophages, wherein M1 macrophages depend on glycolysis and M2 macrophages engage in oxidative phosphorylation, is instrumental in influencing their functional outcomes and lifespan 82, 84. In summary, chronic low-grade inflammation significantly impacts macrophage senescence through various mechanisms, including the activation of inflammatory pathways, metabolic reprogramming, and interactions with other cell types in the tissue microenvironment. These mechanisms lead to a decrease in macrophage functionality, thereby sustaining a cycle of inflammation and tissue injury that is characteristic of aging and diseases associated with older age.

### 3.4 Damage to the DNA repair pathway

The relationship between the DNA repair pathway and macrophage senescence involves a complex interplay that significantly impacts aging and age-related diseases. As key players in the immune response, macrophages are particularly sensitive to DNA damage, which can precipitate cellular senescence. Studies have shown that macrophages with defects in DNA repair pathways, such as those involving the ERCC1-XPF complex, exhibit increased levels of DNA damage. This buildup can initiate various cellular responses, such as the activation of the SASP and additional elements that may exacerbate inflammation in tissues and play a role in age-related diseases 85, 86. Maintaining genomic integrity relies heavily on the DNA damage response (DDR), and when it fails, it can result in oxidative stress. In macrophages, oxidative stress is often a byproduct of persistent DNA damage, which can further drive senescence. In models in which DNA repair is genetically attenuated, such as ERCC1-XPF-deficient mice, there is a notable increase in ROS production. This oxidative stress not only contributes to DNA damage but also activates signaling pathways that promote senescence 87, 88. The interaction between DNA damage and the production of ROS establishes a feedback mechanism that hastens the aging process in macrophages. The mutually reinforcing loop between DNA damage and ROS production accelerates the aging process in macrophages. Moreover, the senescence of macrophages has profound implications for chronic inflammatory conditions, such as atherosclerosis. In atherosclerotic lesions, senescent macrophages tend to accumulate, and their presence is associated with increased inflammation and tissue damage 89. In addition to the immediate impacts of DNA damage on the function of macrophages, during cellular senescence, DNA repair genes are suppressed. This suppression may take place via different pathways, such as the RB/E2F pathway, which oversees the regulation of many DNA repair genes. The downregulation of these genes in senescent macrophages compromises their ability to repair DNA, leading to an increased burden of DNA damage and further promoting the senescent phenotype 90, 91. Furthermore, the role of specific proteins, such as bromodomain-containing protein 4 (BRD4), has been highlighted in the context of macrophage senescence. Inhibiting BRD4 has been demonstrated to impede macrophage aging and decrease lipid buildup in models of inflammation-driven senescence, indicating that focusing on this pathway may present therapeutic possibilities for diseases associated with aging 63, 92.

### 3.5 Mitochondrial dysfunction

Mitochondrial dysfunction and autophagy play significant roles in macrophage senescence, influencing macrophage functionality and contributing to age-related inflammatory disorders. In older macrophages, the integrity of mitochondrial function is diminished, leading to increased vulnerability to excessive leakage of mtDNA and the production of ROS. The buildup of impaired mitochondria has the potential to initiate inflammatory responses by activating pathways such as the NOD-like receptor family pyrin domain containing 3 (NLRP3) inflammasome and the cGAS-STING signaling pathway, which further intensifies inflammation and plays a role in the aging phenotype 93. In senescent macrophages, the PINK1/Parkin-mediated pathway, which is crucial for tagging damaged mitochondria for degradation, is frequently downregulated. This impairment leads to the persistence of dysfunctional mitochondria, further promoting inflammation and cellular senescence 94. Failure to effectively clear damaged mitochondria can initiate a vicious cycle in which mitochondrial dysfunction initiates increased inflammation, exacerbating mitochondrial damage. The process of autophagy, which involves the degradation and recycling of damaged organelles and proteins within cells, is significantly influenced by the aging process. In macrophages, autophagy plays a vital role in controlling inflammation and ensuring cellular balance. Nonetheless, as organisms age, there is a reduction in autophagic flux, resulting in the buildup of impaired cellular components and encouraging senescence. Research indicates that a lack of autophagy in macrophages may result in increased levels of proinflammatory cytokines, such as IL-1β and IL-18, through inflammasome activation 95, 96. Furthermore, the interplay between autophagy and senescence is complex and bidirectional 97. On the one hand, impaired autophagy can lead to cellular senescence by allowing the accumulation of damaged mitochondria and other intracellular components. On the other hand, senescence itself can suppress autophagy through various mechanisms, including the downregulation of autophagy-related genes and the activation of senescence-associated signaling pathways. For example, the mTOR pathway, a key regulator of autophagy, is often dysregulated in senescent macrophages. mTORC1 is a negative regulator of autophagy, and its activation can suppress autophagy initiation.

### 3.6 Telomere shortening

A key way in which the shortening of telomeres influences macrophage senescence is via activation of the DDR. When telomeres shorten, they become dysfunctional, leading to the misrecognition of telomeric DNA as damaged. This triggers DDR signaling pathways and results in cellular senescence. In macrophages, this senescence can manifest as a reduced ability to respond to pathogens and an impaired capacity for tissue repair and regeneration 98. Furthermore, the reduction in telomere length in macrophages is correlated with increased levels of oxidative stress and inflammation. Macrophages obtained from older mice are more vulnerable to oxidants and an increase in ROS, which is linked to telomere shortening. Telomerase knockout (Terc^-/-^) mice exhibit macrophages that share similar characteristics with those of aged mice, such as diminished proliferation reliant on granulocyte macrophage-colony stimulating factor (GM-CSF) and increased oxidative stress. This finding indicates that the loss of telomeres may lead to increased oxidative stress and, consequently, reduced GM-CSF-dependent proliferation in macrophages 99. In addition to the direct impact that telomere shortening has on macrophage aging, evidence suggests that a decrease in telomeres may also affect mitochondrial performance. The interplay between telomere shortening and mitochondrial dysfunction can create a vicious cycle in which telomere attrition leads to mitochondrial impairment, which in turn exacerbates telomere shortening and cellular senescence 100. Furthermore, the regulatory role of specific microRNAs in the context of telomere shortening and macrophage senescence has come to light. For example, miR-146b has been recognized as a possible regulator of macrophage aging, and its expression levels decrease with increasing age. This reduction is correlated with changes in cytokine expression and a decline in mitochondrial metabolic activity, which further contributes to the senescent characteristics of macrophages 27, 101. Telomere shortening significantly impacts macrophage senescence through mechanisms involving DDR activation, increased oxidative stress, and the development of a proinflammatory phenotype.

### 3.7 Cellular metabolic reprogramming

Reprogramming of cellular metabolism significantly impacts the senescence of macrophages, affecting both their function and polarization. Aging macrophages frequently exhibit a transition toward glycolytic metabolism, especially when confronted with inflammatory signals. This metabolic reprogramming is associated with the activation of proinflammatory M1 macrophages, which rely heavily on glycolysis to meet the increased energy demands during inflammation. However, this reliance on glycolysis can lead to metabolic inflexibility, resulting in an inability to switch to oxidative metabolism when needed, which is crucial for resolving inflammation and promoting tissue repair 84, 102. A study showed that Tp47 stimulates the NLRP3 inflammasome, which results in increased phosphorylation of EIF2AK2 and the subsequent release of proinflammatory cytokines associated with senescence, ultimately leading to inflammatory senescence in macrophages 103.

## 4. Macrophage Senescence and Cancer Progression

**Figure 3:**
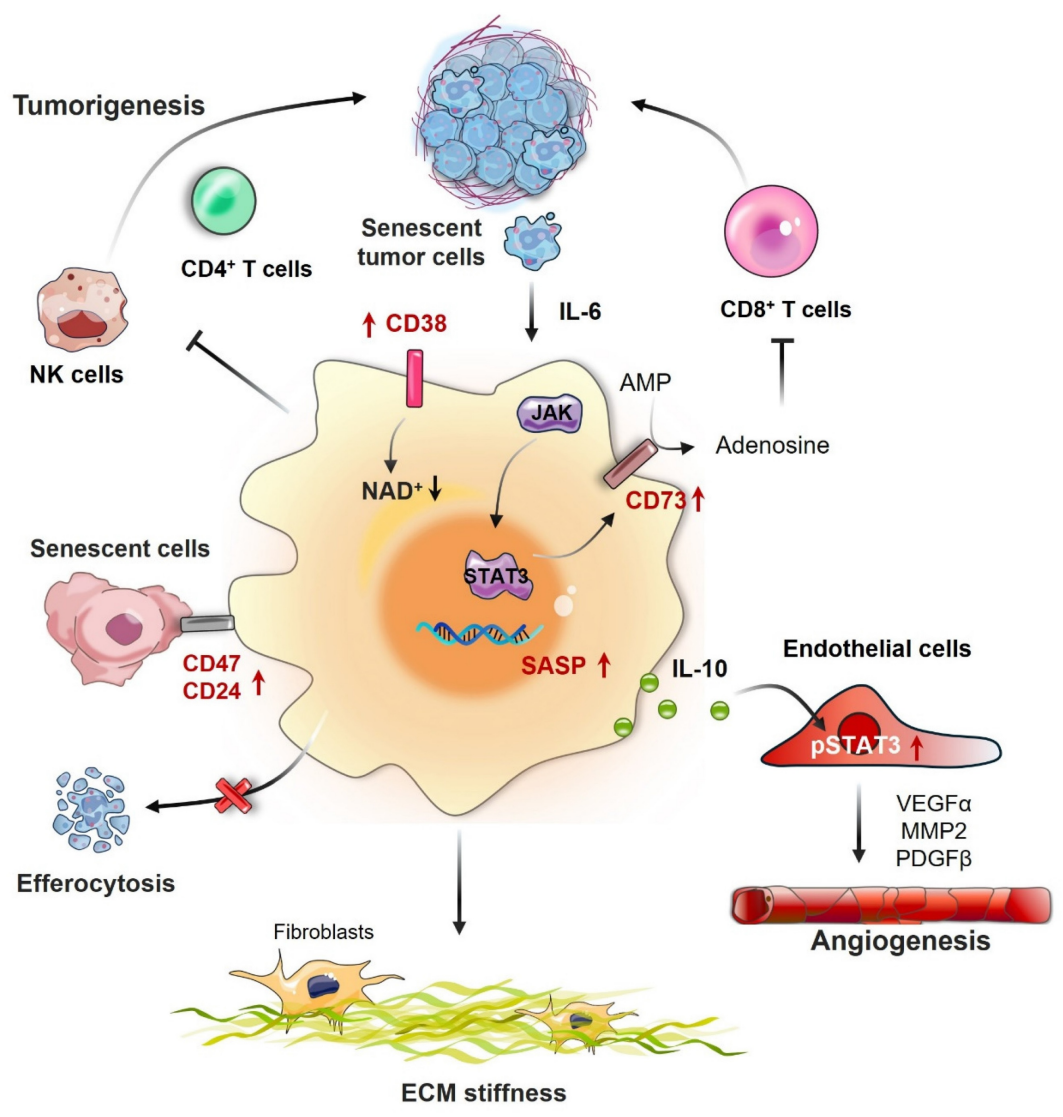
** Impact of macrophage senescence in the TME.** Senescent macrophages significantly influence the tumor microenvironment (TME) through multiple pathways. They release an array of proinflammatory cytokines, such as IL-6 and TNF-α, which promote tumor cell proliferation and survival. By modulating immune cell interactions, senescent macrophages can attract regulatory T cells (Tregs) and myeloid-derived suppressor cells (MDSCs), thereby establishing an immunosuppressive milieu that hinders antitumor immune responses. Additionally, they contribute to angiogenesis by secreting vascular endothelial growth factor (VEGF), increase extracellular matrix (ECM) stiffness through the action of matrix metalloproteinases (MMPs), and facilitate immune evasion by downregulating major histocompatibility complex (MHC) molecules on tumor cells, making them less recognizable to the immune system.

The senescence of macrophages has a crucial impact on the emergence and advancement of tumors, affecting both the TME and immune responses. Macrophages that have undergone senescence may aid in the initiation and progression of cancer through multiple mechanisms, mainly by releasing proinflammatory cytokines and modifying interactions among immune cells (Fig. 3). As shown in Table 2, studies on senescent macrophages have been conducted across multiple types of cancer, highlighting their diverse and complex roles in tumor biology and the immune landscape.

### 4.1 Tumor escape and immunosuppression by senescent macrophages

Although macrophages usually play a crucial role in eliminating senescent cells and sustaining tissue balance, their effectiveness may be greatly compromised throughout the senescence process. This impairment affects their ability to recognize and eliminate tumor cells, potentially leading to augmented tumor escape and growth 104. Tumor-derived factors can induce senescence in macrophages, promoting the expression of immunosuppressive molecules such as arginase-1. The upregulation of these molecules can impair T-cell function, allowing tumor growth. Senescence-like macrophages also exhibit increased expression of CD38, a marker associated with metabolic dysfunction in senescent cells, which can further impair T-cell function and promote tumor escape mechanisms 26. Haston et al*.* identified a population of senescent macrophages with protumorigenic activities in KRAS-driven murine models of lung adenocarcinoma. These cells exhibit a unique molecular signature that is conserved in the physiologically aged lung. This study also revealed that macrophages express senescence markers in human premalignant lung lesions. Critically, they demonstrated that senescent macrophages in both tumor-bearing and naturally aged lungs share molecular similarities and that their ablation reduces the tumor burden by enhancing antitumor immunosurveillance 68. This finding underscores the dual role of senescent macrophages in bridging age-related immune dysfunction and cancer susceptibility. Duong and colleagues reported that mesothelioma tumors developed more rapidly in older mice than in their younger counterparts, which was associated with a greater presence of TAMs within the TME. The team discovered that the proliferative capacity of macrophages from older mice varied and was characterized by increased proliferation of bone marrow macrophages and reduced proliferation of splenic macrophages throughout the process of tumor growth. These findings suggest that aging enhances the supply of macrophages to the tumor site, potentially exacerbating tumor progression. Additionally, the depletion of macrophages via the F4/80 antibody considerably reduced tumor growth in both young and older mice. It also enhanced the effectiveness of IL-2/anti-CD40 immunotherapy in older mice, resulting in greater tumor regression and improved survival rates. In contrast, macrophage depletion in young mice impaired the effectiveness of immunotherapy, indicating that macrophages are essential for antitumor immune responses in younger hosts but become dysregulated and detrimental in elderly individuals 75.

### 4.2 Promotion of angiogenesis by senescent macrophages

Research has demonstrated that macrophages in a senescent state can produce various proangiogenic factors, which play a significant role in the angiogenic process 41, 105. These macrophages, resembling senescent macrophages, increase the level of pSTAT3 in endothelial cells, which leads to the direct interaction of pSTAT3 with the promoters of VEGFA, MMP2, and PDGFB, thus promoting their synthesis and facilitating angiogenesis. Additionally, senescent macrophages participate in a feedback mechanism that further enhances angiogenesis. They not only secrete VEGF but also release other factors that can attract more macrophages and endothelial cells to the tumor environment, consequently intensifying the angiogenic response 106.

### 4.3 Extracellular matrix remodeling

A crucial function of senescent macrophages is their involvement in the remodeling of the extracellular matrix (ECM), which can affect the behavior of tumor cells, especially invasion and metastasis. As individuals age, the accumulation of senescent cells, including macrophages, results in changes in the tissue microenvironment. These alterations are typically marked by the secretion of diverse factors that fuel a proinflammatory state, thereby modifying the composition and mechanical properties of the ECM. Senescent macrophages are known to release greater amounts of MMPs, which are enzymes responsible for breaking down different components of the ECM. This breakdown results in a more favorable environment for tumor cells, facilitating their movement and infiltration into adjacent tissues 107. The mechanical characteristics of the ECM, including its stiffness, play a vital role in influencing cell behavior. Senescent macrophages can influence the rigidity of the ECM through their secretions, potentially leading to an increase in tissue stiffness. This change in mechanical properties can enhance the invasive capabilities of tumor cells, as many cancer cells are sensitive to the stiffness of their surrounding environment 108. Increased stiffness of the ECM can facilitate the movement and spread of cancer cells by modifying cell signaling pathways and increasing the expression of genes associated with invasion 109.

### 4.4 Metabolic regulation in the TME

A crucial function of senescent macrophages in the TME is their capacity to affect the metabolic reprogramming of cancer cells by either producing or consuming a variety of metabolites. Senescent cells are known to produce SASP, which includes a variety of cytokines, such as IL-6 and IL-8. These cytokines can stimulate tumor cell proliferation and survival while also promoting a chronic inflammatory microenvironment that supports tumor development. The presence of these cytokines can precipitate metabolic alterations in both macrophages and tumor cells, creating a reciprocal feedback loop that exacerbates tumor growth 84, 102. Senescent macrophages can influence the availability of nutrients and metabolites within the TME. Tumor cells often compete for resources such as glucose, amino acids, and glutamine, which can induce metabolic stress in immune cells, including macrophages. This competition can result in a metabolic shift in macrophages, where they may adapt their metabolism to support tumor growth rather than their typical immune functions. The buildup of adenosine within the TME, which is frequently aided by macrophages that express ectonucleotidases such as CD73, has the potential to inhibit the antitumor functions of different immune cell types, notably cytotoxic T cells 110. This accumulation of adenosine can further promote tumor cell survival and proliferation. The metabolic reprogramming of macrophages can lead to changes in their polarization state. Aging macrophages can transform into a protumor M2-like phenotype, which is characterized by increased secretion of anti-inflammatory cytokines and a diminished ability to present antigens to T cells. This transition not only suppresses the immune response to target tumors but also fosters a metabolic environment that facilitates tumor proliferation 78. Aging macrophages release proinflammatory cytokines, vie for resources, and adjust their metabolic pathways, which can foster an environment conducive to tumor growth and persistence.

## 5. Therapeutic Implications of Macrophage Senescence in Cancer

**Figure 4:**
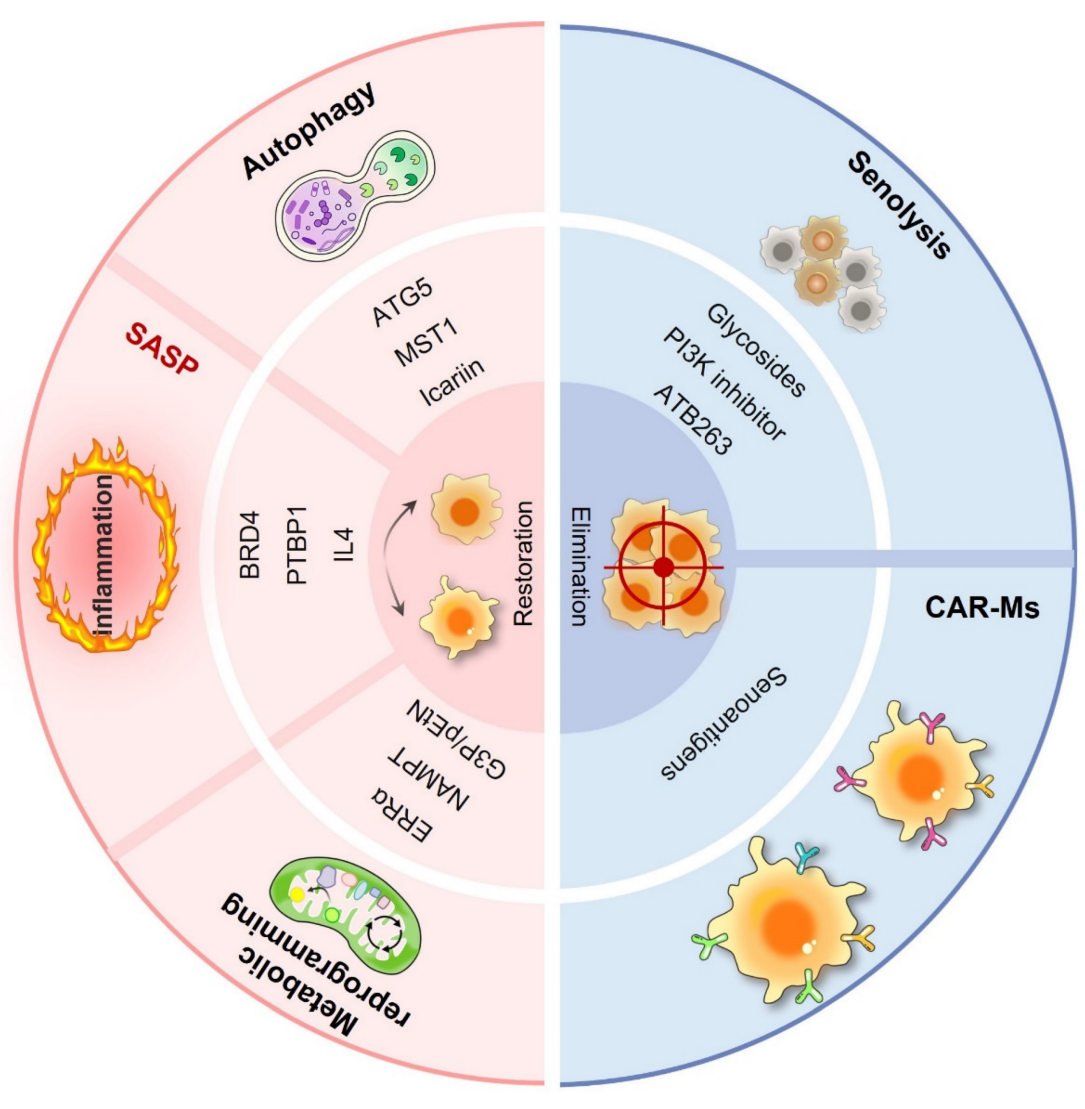
** Macrophage senescence-targeted therapeutic strategies in cancers.** Macrophage senescence-targeted therapy encompasses a diverse range of approaches aimed at reversing senescence and eliminating senescent macrophages. These include senolytic drugs designed to selectively induce apoptosis in senescent cells, such as navitoclax and the dasatinib/quercetin combination, which have shown promise in preclinical models by reducing tumor burden and enhancing treatment efficacy. CAR-M therapy represents an innovative strategy in which macrophages are engineered to express chimeric antigen receptors (CARs) that specifically target senescent cells within the TME.

In the realm of cancer therapeutics, conventional treatments have made progress but still face numerous challenges. Immunotherapy, despite offering new hope, exhibits variable efficacy, partly owing to the complexity of the TME. In this intricate environment, macrophages, especially in senescent conditions, are crucial in influencing tumor advancement, growth, and response to treatment. Senescent macrophages secrete a plethora of inflammatory cytokines and chemokines, creating an immunosuppressive milieu that undermines immune surveillance and diminishes the effectiveness of immunotherapies. They may also affect the sensitivity of tumor cells to chemotherapeutic agents, potentially facilitating drug resistance in tumor cells. However, the phenomenon of macrophage senescence presents novel therapeutic targets and clinical applications (Fig. 4). As illustrated in Table 3, a variety of therapeutic strategies have been proposed. These senescence-targeted therapeutic strategies offer new avenues for cancer treatment and hold promise for improving patient outcomes in future clinical applications.

### 5.1 Impact on existing cancer therapies

Senescent macrophages exhibit altered functionality that can impact the efficacy of various cancer treatments, including immunotherapy and chemotherapy. Although senescent macrophage-derived SASP factors can create an immunogenic environment that may initially support antitumor immunity, the prolonged presence of SASP factors can lead to immune evasion, complicating the therapeutic landscape 111, 112. On the other hand, the senescence of cancer cells induced by chemotherapy, which is influenced by the SASP element known as macrophage colony-stimulating factor (M-CSF), encourages the shift of macrophages toward the M2 phenotype. This shift creates an immunosuppressive microenvironment that aids tumor cells in evading immune surveillance and contributes to chemotherapy resistance 113. The relationship between aging macrophages and cancer cells highlights the intricate nature of the TME, where senescent macrophages may inadvertently support tumor growth and metastasis, counteracting the intended effects of cancer therapies. Within the realm of immunotherapy, especially involving agents aimed at enhancing T-cell responses, the existence of senescent macrophages can prove harmful. Evidence from studies indicates that macrophages sourced from older mice display an intensified reaction to factors derived from tumors, resulting in increased release of immunosuppressive cytokines such as TGF-β and IL-10, which can hinder antitumor T-cell activity. This environment of cytokines has the potential to guide the ability of dendritic cells to promote T-cell tolerance, which in turn reduces the effectiveness of immunotherapy approaches. Additionally, the imbalance of macrophages observed in older individuals, marked by increased numbers and altered growth patterns, indicates that focusing on these cells may improve the success of immunotherapy for elderly cancer patients 75. The transition of macrophages to a senescent state result in the secretion of immunosuppressive factors, polarization toward a protumorigenic phenotype, and the establishment of a suppressive immune microenvironment. These changes can hinder the effectiveness of cancer treatments and promote tumor recurrence.

### 5.2 Potential therapeutic targets and clinical applications of macrophage senescence

#### 5.2.1 Therapeutic targets in macrophage senescence

##### 5.2.1.1 Inhibition of the SASP

Senescent macrophages can secrete a variety of SASP factors that not only perpetuate their own senescence but also affect the behavior of surrounding cells, initiating a deleterious cycle of inflammation and tissue damage. To modulate senescent macrophages and suppress the SASP, several strategies can be employed. One such strategy involves targeting the signaling pathways that drive SASP expression. BRD4 has been identified as a key regulator of the SASP in macrophages. Inhibition of BRD4 has been shown to prevent the aging of macrophages and lipid accumulation in senescent macrophages induced by LPS, thereby reducing SASP expression and its associated inflammatory effects 63. These findings suggest that pharmacological agents targeting BRD4 or analogous signaling pathways could be effective in modulating the SASP and improving macrophage function during aging. Another potential strategy is the use of bioactive compounds that can modulate autophagy and inflammation. Icariin, an active component present in traditional Chinese herbal medicines, can activate autophagy and has anti-inflammatory effects on aging macrophages. This substance not only enhances the osteogenic capacity of aged bone marrow mesenchymal stem cells but also lowers the levels of SASP factors, suggesting its promise as a therapeutic agent for addressing inflammation associated with senescence 114.

The importance of miRNAs in modulating the SASP and the senescence of macrophages is receiving increasing interest. Certain miRNAs have been shown to modulate the expression of SASP components and influence the senescence process. For example, miR-340-5p has been shown to promote cellular senescence and enhance sensitivity to senolytic compounds, suggesting that targeting such miRNAs could provide a novel approach to regulate the SASP and mitigate the effects of senescent macrophages 115. Zhou et al*.* reported that a lack of IL-4 signaling hastens the aging process, whereas the IL-4-STAT6 pathway provides protection to macrophages against senescence. When STAT6 is deficient, nuclear DNA is released into the cytoplasm, which activates the cGAS-STING pathway, resulting in enhanced tissue inflammation and overall aging in the organism 35. Several strategies are emerging for this purpose. One promising approach is to enhance IL-4 signaling, either through gene therapy or pharmacological means, to increase STAT6 activation and subsequent DNA repair pathways. An alternative approach focuses on the cGAS-STING pathway, which is activated due to DNA damage and plays a role in inflammation linked to senescence.

##### 5.2.1.2 Increased autophagy

Autophagy is essential for preserving the cellular balance through the degradation of damaged organelles and proteins. Research has indicated that the efficiency of autophagic processes is significantly reduced in older macrophages, which results in compromised functions such as phagocytosis and heightened inflammatory responses 116, 117. A reduction in autophagic function correlates with a change in macrophage polarization toward a proinflammatory M1 phenotype, intensifying inflammation and playing a role in the aging process. Therefore, enhancing autophagy in macrophages could help restore their functionality and counteract the aging process. Autophagy-related protein 5 (ATG5) is critical for autophagy initiation, and its deficiency has been linked to decreased autophagic activity and increased senescence markers in macrophages. By promoting the expression or activity of genes such as *Atg5*, enhancing autophagy in senescent macrophages may be feasible. This could increase their ability to clear damaged organelles and proteins, reduce inflammation, and restore normal immune function. Moreover, certain compounds that can stimulate autophagy have been identified. For example, quercetin, a natural flavonoid, has been shown to increase autophagy in macrophages, resulting in reduced lipid accumulation and senescence 118.

##### 5.2.1.3 Modulating metabolic pathways

One of the critical metabolic pathways that regulate cellular senescence is the mevalonate pathway, which is responsible for the biosynthesis of cholesterol and other polyisoprenoids. This pathway is considered a positive regulator of senescence in typical human cells. In particular, activating the mevalonate pathway may result in the buildup of cholesterol, which enhances the transcriptional activity of the estrogen-related receptor (ERR). This activation is linked to mitochondrial dysfunction, elevated production of ROS, and damage to DNA, which ultimately results in p53-dependent senescence 119. Disruption of this pathway has been associated with a decrease in markers of senescence, indicating that targeting the biosynthesis of cholesterol might be an effective strategy for delaying macrophage senescence. Furthermore, the importance of NAD metabolism in the context of senescence deserves attention. With aging, NAD levels decrease, and this decline corresponds with the activation of senescence pathways. Senescent cells display increased levels of nicotinamide phosphoribosyltransferase (NAMPT), the enzyme responsible for the rate-limiting step in the biosynthesis of NAD. Paradoxically, the increase in NAMPT does not correlate with increased NAD levels, indicating a dysfunction in NAD metabolism during senescence 120. Increasing NAD levels through supplementation with NAD precursors has been shown to prevent cellular senescence and improve metabolic health, indicating that strategies aimed at restoring NAD levels could be beneficial for maintaining macrophage function and delaying senescence. Tighanimine et al*.* employed dynamic profiling of the transcriptome and metabolome of human fibroblasts and demonstrated that a homeostatic mechanism responsible for the buildup of G3P (glycerol-3-phosphate) and pEtN (phosphoethanolamine) links lipid metabolism to the expression of senescence-related genes. This mechanism is regulated through the p53-mediated activation of glycerol kinase and the posttranslational inactivation of phosphate cytidylyltransferase 2, which is associated with ethanolamine. This process encourages the accumulation of triglycerides within lipid droplets and triggers the senescence gene expression program. Conversely, the scavenging of G3P and pEtN through G3P phosphatase and ethanolamine-phosphate phospho-lyase acts in a senomorphic way, reducing their accumulation and potentially mitigating senescence 121. These findings suggest that targeting metabolic pathways involved in G3P and pEtN accumulation could offer therapeutic strategies to delay senescence in macrophages.

#### 5.2.2 Clinical applications of targeting macrophage senescence in cancer therapy

##### 5.2.2.1 Senolytic drugs

Senolytic medications, which specifically trigger apoptosis in aging or senescent cells, have attracted considerable interest in the context of cancer therapy, especially given their ability to influence the TME and the immune response. Recent research has indicated that these senolytic agents can successfully target senescent cancer-associated fibroblasts (CAFs) and macrophages present in the TME. For example, the combination of dasatinib and quercetin significantly decreases the number of senescent CAFs and alters the release of proinflammatory cytokines, including IL-6, which is linked to tumor progression 122. These findings were observed in both cell culture and mouse models, such as in studies using primary mouse dermal fibroblasts and p16-3MR mice. These findings demonstrate that senolytic treatment can help address the detrimental impacts of senescent macrophages and CAFs, potentially improving the effectiveness of standard cancer treatments. Although there are worries about the possible dangers linked to the elimination of senescent cells, especially concerning compromised tissue repair, research also indicates that judicious use of these therapies could reduce those risks 123. Demonstrating the safety, efficacy, and tolerability of senolytic drugs in human subjects presents a challenge, particularly because of the diverse characteristics of senescent cells found in various tissues and under different conditions 124. In the context of clinical application, the identification of specific senolytic agents that can effectively target senescent macrophages without adversely affecting normal immune function is crucial. For example, cardiac glycosides have been recognized as a category of senolytic agents capable of selectively targeting and removing senescent cells, such as macrophages, by leveraging their distinctive weaknesses 125. This method has the potential to improve the immune response against tumors while mitigating the protumor effects associated with senescent macrophages. Additionally, combining senolytic therapies with standard cancer treatments such as chemotherapy and radiotherapy presents a promising tactic. Treatment-induced senescence (TIS) can manifest in both cancerous and healthy cells after therapy, resulting in complicated interactions that may affect the results of treatment. By targeting senescent cells in the TME, senolytic drugs could help alleviate some of the negative consequences associated with TIS, such as chronic inflammation and tumor relapse 126.

Navitoclax (ABT-263), a senolytic drug, has shown promise in clinical trials targeting senescent cells. Its ability to selectively clear senescent cells has been explored under various age-related conditions. Zhang et al*.* revealed that ABT-263 treatment improved the survival rate of aged mice with sepsis by increasing the expression of Trem-2 receptors and inducing autophagy. This effect occurred through the inhibition of the interaction between B-cell lymphoma-2 (Bcl-2) and Beclin-1, which in turn facilitated autophagy and improved the phagocytic capabilities of macrophages 127. Additionally, Furukawa et al*.* demonstrated in a separate study that ABT-263 can lower the levels of inflammatory cytokines in aged gingival fibroblasts, indicating its potential role in addressing age-related alterations in the gingiva 128. In clinical trials, Navitoclax has been used to treat hematological malignancies, where it targets Bcl-2 family proteins to induce apoptosis in cancer cells 129, 130. This mechanism of action also makes it a potential candidate for managing age-related diseases characterized by increased senescence, such as cardiovascular diseases and neurodegenerative disorders. By reducing the burden of senescent cells, Navitoclax may help alleviate chronic inflammation and improve tissue function, thereby extending health span and potential lifespan.

##### 5.2.2.2 Chimeric antigen receptor macrophage (CAR-M) therapy

Recent progress has explored the use of genetically modified macrophages, known as chimeric antigen receptors (CARs), to improve antitumor responses by focusing on senescent cells found within the TME. CAR-M therapy leverages the unique phagocytic capabilities of macrophages, which are the most abundant immune cells infiltrating solid tumors. These engineered macrophages can be designed to express CARs that specifically target antigens present on senescent cells, thereby promoting their clearance 131. In preclinical studies, CAR-M have demonstrated the ability to home to various solid tumors, including breast, ovarian, pancreatic, and colorectal cancers 132. They effectively phagocytose cancer cells and can alter the TME by transforming immunosuppressive M2 macrophages into proinflammatory M1 macrophages. This transformation is crucial, as it enhances the overall antitumor immune response, potentially leading to improved patient outcomes 133, 134. Moreover, the synergistic application of CAR-M therapy in conjunction with established treatments such as chemotherapy and immune checkpoint inhibitors has become a promising approach to enhance therapeutic results. Reports indicate that selective class IIa HDAC inhibitors can influence the macrophage phenotype, resulting in decreased tumor burden and increased survival rates in mouse models of breast cancer 135. By specifically targeting senescent cells, CAR-M could help mitigate the adverse effects of SASP factors and restore immune function within the TME. This targeted approach may not only increase the efficacy of cancer treatments but also reduce the side effects associated with nonspecific therapies. Furthermore, advancements in CAR-M capable of effectively targeting senescent cells may pave the way for novel therapeutic strategies to combat age-related diseases and various disorders characterized by cellular senescence. As research progresses, several CAR-M therapies, such as CT-0508 and MCY-M11, are undergoing clinical trials and have shown promising safety profiles and tolerability in early-phase testing 136, 137. The identification of specific senescence-associated surface antigens (senoantigens) is critical for the effective implementation of CAR-M therapies in clinical settings.

## 6. Clinical Challenges and Future Directions in Macrophage Senescence Research

### 6.1 Clinical challenges

Exploring macrophage senescence poses numerous clinical difficulties due to the complexities of the immune system, the diversity of macrophage populations, and the intricate characteristics of aging and related diseases. Key hurdles in this field include the following: (1) Heterogeneity of macrophage populations: Macrophages are not a uniform cell type, differing in their origin, tissue localization, and activation status. This diversity complicates the characterization of senescent macrophages, as different subsets may exhibit distinct senescence markers and functional properties. Aging tends to increase the prevalence of certain macrophage subsets, such as CCR2^+^ macrophages, which express higher levels of proinflammatory cytokines than their younger counterparts do 138. This inherent variability makes it difficult to establish a clear definition of what constitutes a senescent macrophage across different tissues and conditions. (2) Identification of senescence markers: The identification of senescent macrophages relies on markers such as p16INK4a and SA-galactosidase activity. However, these markers can also be expressed in activated macrophages, leading to misclassification 61. Furthermore, the expression levels of these markers are also influenced by the microenvironment and inflammatory cues, which increases the difficulty of their identification. (3) Influence of the TME: In cancer settings, TAMs may assume an immunosuppressive phenotype that hampers the activity of antitumor T cells. Aging may exacerbate this immunosuppressive effect, with age-related alterations in macrophage function potentially obstructing effective tumor immunity 75. Understanding how senescence in macrophages contributes to tumor progression and immune evasion is critical yet remains poorly understood. (4) Functional impairments: Senescent macrophages often exhibit impaired functions, such as reduced phagocytic capacity and altered cytokine production profiles. These functional changes can compromise their ability to respond to infections or clear damaged cells, a critical concern in age-related diseases 116. Evaluating these functional impairments in clinical settings poses challenges, as traditional assays may not fully capture the nuanced changes in macrophage behavior associated with senescence. (5) Therapeutic implications: Focusing on senescent macrophages as therapeutic approaches for ailments such as cancer and age-related disorders raises concerns regarding possible adverse effects. Although the removal of senescent cells could enhance tissue function, it might also interfere with helpful macrophage populations that are essential for tissue repair and regeneration 138. The development of strategies that selectively target deleteriously senescent macrophages without affecting their beneficial counterparts is a substantial therapeutic challenge. Additionally, the paucity of elderly participants in clinical trials and the observed reduced efficacy of immunotherapies in this population compared with younger individuals present significant hurdles. These factors can influence the safety and tolerability profiles of immunotherapies, potentially increase the risk of adverse events and complicating the interpretation of treatment outcomes.

### 6.2 Future directions

Research on macrophage senescence is an evolving field that has significant implications for understanding aging and age-related diseases. Future directions in this area can be categorized into several key themes: (1) Mechanistic elucidation of senescence: A more thorough comprehension of the molecular mechanisms that drive macrophage senescence is essential. This involves unraveling the functions of important signaling pathways, including p53/p21 and Rb/p16INK4a, in the control of cellular senescence, as well as exploring how they interact with stressors such as oxidative stress and inflammation to identify the factors initiating macrophage senescence. (2) Functional profiling of senescent macrophages: Future studies should focus on characterizing the functional metamorphoses that senescent macrophages undergo. This includes a thorough examination of their cytokine secretion patterns, phagocytic capacity, and response to pathogens. Understanding how these functional changes fuel the inflammatory microenvironment associated with aging and chronic diseases is essential. (3) Therapeutic targeting of senescent macrophages: The deleterious impacts of senescent macrophages in diseases ranging from cancer to chronic inflammation necessitate the development of targeted therapeutic modalities. This could involve the exploration of senolytic agents capable of eliminating senescent macrophages or modulating their function to restore homeostasis. (4) Impact of aging on macrophage plasticity: Understanding how the aging process affects the plasticity of macrophages, particularly their ability to polarize into M1 or M2 phenotypes, is critical. Identifying the environmental cues that influence this plasticity in an aging context could pave the way for interventions to increase immune responses in elderly individuals. (5) Clinical implications and biomarkers: Identifying biomarkers of macrophage senescence that can be used in clinical settings to assess the health status of individuals, particularly in aging populations, is another important direction. This could aid in the early detection of age-related diseases and the monitoring of therapeutic responses. Moreover, there is a need for more clinical trials tailored to target immunosenescent cells. These trials could test novel therapeutic strategies, such as senolytic drugs or engineered immune cells, to increase the effectiveness of immunotherapy in elderly patients. Ultimately, examining the impact of environmental elements such as nutrition, physical activity, and contact with pollutants on macrophage aging and performance may yield valuable information for developing preventive approaches for age-related diseases.

## 7. Conclusion

This review offers an extensive examination of how macrophage senescence impacts cancer biology, emphasizing its diverse effects on tumor development. Senescent macrophages, which are characterized by changes in cytokine secretion, diminished phagocytic function, and metabolic reprogramming, contribute to tumor proliferation while also facilitating immune escape and the formation of new blood vessels. Understanding these mechanisms is crucial for the development of novel therapeutic strategies aimed at addressing macrophage senescence. Despite the challenges posed by the heterogeneity of macrophage populations and the intricate dynamics of the TME, the study of macrophage senescence offers promising avenues for intervention. Clinically, the identification of senescent macrophages as key players in tumor progression and therapy resistance highlights the potential for developing targeted therapies. Senolytic agents and CAR-M therapies represent innovative approaches that could enhance the effectiveness of existing cancer treatments. Future research should focus on delineating the specific functions of various macrophage subsets during senescence, exploring their crosstalk with other cellular components, and advancing clinical studies to validate targeted therapies against senescent macrophages. By focusing on these aspects, there is a possibility to enhance cancer therapies and boost patient results, utilizing the unique characteristics of macrophage aging in the TME.

## Figures and Tables

**Table 1:** Characteristics of macrophages during aging.

Characteristics	Feature	Specific manifestations	Ref.
Functional changes	Phagocytic capacity decline	Aging impairs macrophage phagocytosis, with young macrophages also showing reduced function in the aged microenvironment.	53
	Impaired pathogen clearance	Young BMDMs effectively clear *S. pneumoniae* via LC3-associated phagocytosis, while aged BMDMs exhibit deficiencies, producing more pro-inflammatory cytokines.	54
	Reduced clearance of apoptotic cells	Aging reduces MerTK levels on macrophages, exacerbating pulmonary inflammation post I/R injury.	55
Metabolic features	Decreased metabolic activity	Senescent macrophages show reduced metabolic rate and energy pathway efficiency, affecting immune functions.	29
	ROS accumulation	Increased NOX4 expression and PGE2-EP2 signaling lead to energy-deficient states and inflammation in aging macrophages.	57, 58
	Metabolic reprogramming	A shift to glycolysis in M1 macrophages increases ROS and cytokines, worsening senescence.	100
Morphological and phenotypic features	Cellular morphological changes	Senescent macrophages enlarge and exhibit nuclear and organelle alterations.	61
	Biochemical phenotypic heterogeneity	SR-FTIR reveals dynamic lipid and protein changes during macrophage senescence.	62
	Protein expression changes	Upregulated proteins like p53, p21, and γ-H2A.X indicate DNA damage, with RNA sequencing showing metabolic and signaling pathway alterations.	63
Role in chronic diseases	Promotion of inflammation and fibrosis	Senescent macrophages link obesity, inflammation, and metabolic disorders through their accumulation and functional changes.	64, 65
	Impact on TME	These macrophages support tumor growth and metastasis by secreting cytokines and growth factors, with higher infiltration in elderly patients' tumors.	67, 73
Changes in cell‒cell interactions	Altered interactions with T cells	Aging induces immunosuppressive environments in the TME, hindering T-cell activity.	74, 75
	Modified interactions with stem cells	Impaired MSC function in aged conditions delays inflammation resolution and tissue regeneration.	74, 77

**Table 2:** Research on macrophage senescence in different types of cancer.

Cancer type	Research findings	Ref.
Lung cancer	Senescent alveolar macrophages accumulate in the early stages of lung tumorigenesis, characterized by increased expression of p16INK4a and CXCR1, and are capable of suppressing cytotoxic T-cell responses.	14
Colorectal cancer	Senescent tumor cells secrete CXCL12 and CSF1, promoting the differentiation of monocytes into M2 macrophages, thereby suppressing CD8^+^ T-cell activation and promoting tumor progression.	67
Thyroid cancer	Senescent thyroid cells and thyroid cancer cells induce M2-like macrophage polarization in human monocytes through a prostaglandin E2 (PGE2)-dependent mechanism.	105
Hepatocellular carcinoma	Allicin regulates Sestrin2 ubiquitination to affect macrophage autophagy and senescence, thereby inhibiting the growth of hepatoma cells. Additionally, senescent macrophages in hepatocellular carcinoma are activated through a Bcl3-dependent mechanism, promoting hepatocarcinogenesis.	69, 70
Gastric cancer	Aurora kinase inhibitors induce senescence in macrophages within gastric cancer organoids, characterized by increased expression of senescence markers and the secretion of MCP-1/CCL2, which recruits and induces M2 polarization, suppressing the innate immune response to cancer cells.	32

**Table 3:** Therapeutic strategies targeting senescent macrophages in cancer models.

Treatment	Treatment method/mechanism	Ref.
Senolytic drugs	Navitoclax (ABT-263): This drug is a Bcl-2 family inhibitor that targets anti-apoptotic proteins such as BCL-2, BCL-W, and BCL-xL. By inhibiting these proteins, Navitoclax promotes apoptosis in senescent cells, making it effective in eliminating cells that have entered a state of senescence due to stress or damage. Dasatinib and Quercetin: Dasatinib is a tyrosine kinase inhibitor that can induce apoptosis in senescent cells, while Quercetin is a flavonoid that has antioxidant properties and can also promote cell death in senescent cells. Ouabain: It sensitizes senescent cells to apoptosis through the induction of the pro-apoptotic protein NOXA. HSP90 Inhibitors: Inhibition of HSP90 can lead to the degradation of client proteins, thereby inducing apoptosis in senescent cells. N-myristoyltransferase inhibitors (NMTi): These inhibitors have been shown to selectively eliminate senescent cells by disrupting Golgi function and autophagy, leading to apoptosis.	126, 139-141
CAR-macrophages (CAR-Ms) therapy	Employing genetically engineered macrophages (CAR-Ms) to target senescent cells within the TME, promoting their clearance.	131, 133, 142, 143
Inhibition of SASP	Modulating senescent macrophages by inhibiting the expression of SASP to reduce inflammation and tissue damage.	63, 144
Enhancing autophagy	Restoring the function of senescent macrophages by promoting autophagy to reduce inflammation and restore normal immune function.	96, 116-118
Modulating metabolic pathways	Regulation of cholesterol biosynthesis and NAD metabolism, to delay macrophage senescence. Modulating the AKT2 pathway could improve macrophage function in clearing senescent cells.	108, 119, 120
Immune modulation	Delaying macrophage senescence by enhancing IL-4 signaling or targeting the cGAS-STING pathway. CD47/SIRPα Axis: CD47 acts as a “don't eat me” signal when it binds to the SIRPα receptor on macrophages, inhibiting their phagocytic activity. Blocking this interaction can enhance macrophage-mediated destruction of cancer cells, making it a promising target for immunotherapy.	35, 145
